# Supplementary material for: A film-lever actuated switch technology for multifunctional, on-demand, and robust manipulation of liquids
Source: Nat Commun. 2022 Aug 20;13:4902. doi: 10.1038/s41467-022-32676-4 (PMC9391643; doi:10.1038/s41467-022-32676-4)
Supplement: Supplementary file 1 — Supplementary Information [file 41467_2022_32676_MOESM1_ESM.pdf]

Supplementary Information

A film-lever actuated switch technology for multifunctional, on-demand and robust manipulation of liquids

Chao Liang[1], Zihang Yang[2], Hanqing Jiang[1*]

[1]School of Engineering, Westlake University, Hangzhou, 310024, China

[2]Hangzhou Vantronics Biotechnology Ltd., Hangzhou, 311100, China

*Email: hanqing.jiang@westlake.edu.cn

Content of supplementary materials

Supplementary Note. Theoretical analysis for the FAST-POCT platform.

Figure S1. FAST-POCT platform components and illustration for assembly.

Figure S2. Schematic diagram for the dimensions of FAST-POCT.

Figure S3. Schematic diagram for theoretical analysis of FAST.

Figure S4. Pictures of the testing system for the fluid flowing behavior.

Figure S5. Images of the surface roughness comparison of a, 3D printing material and b, PMMA material.

Figure S6. Bubble mixing performance.

Figure S7. Temperature profile for the testing process.

Figure S8. The results for IBV PCR testing using FAST-POCT platform and benchtop platform with FAST extracted RNA samples

Figure S9. FAST-POCT instrument.

Figure S10. Illustration of the FAST-POCT testing module.

Figure S11. Flow chart of the working principles of the FAST-POCT instrument.

Figure S12. Comparison of PCR performance for shaking mixing, bubble mixing and without mixing as a control group.

Figure S13. Pictures of a, nasal swab collection site and b, the collected nasal swab.

Supplementary Table 1. Material cost estimate of the FAST-POCT system.

Supplementary Table 2. Comparisons among some POCT PCR studies and

technologies.

Supplementary Table 3. Clinical sample information.

Movie S1. Demonstration of the lever movement of the FAST-POCT platform.

Movie S2. Multifunctional dispensing demonstration, including cascaded, simultaneous, sequential and selective dispensing modes.

Movie S3. Robustness test video using a capillary-based device as a comparison.

Movie S4. On-demand releasing test video using a capillary-based device as a comparison.

Movie S5. Flow behavior testing video with the liquids of different properties.

Movie S6. Working procedure video of the FAST-POCT platform.

Movie S7. Thermal imaging video for one thermocycle.

Movie S8. Wax sealing process of the FAST-POCT platform using a non-wax device as a comparison.

Supplementary Note

Theoretical analysis for the FAST-POCT platform. It can be seen from **Figure S2** that when no force is applied, the equilibrium equation can be expressed as:

$$\sum \mathrm{M_A} = 0 \tag{1}$$

$$\mathrm{F}_s l = T_x^* \tag{2}$$

where ΣM_A is the resultant moment about the hinge point A, F_s is the sealing force at the block point B, l is the distance between the block and the hinge, and T_x^* denotes the torque at the hinge generated by lever. The sealing force depends on the interference fit.

To open a gap between the block and the lever to let the liquid flow, the exerted torque about the hinge needs to overcome T_x^*. In other words, when a uniformly distributed load q (= P_cS) is applied with P_c as the critical pressure value and S as the area of the lever, the torque generated by q should be greater than T_x^*, i.e.,

$$\frac{\mathrm{qL}}{2} > T_x^* \tag{3}$$

which gives the critical pressure $P_c \geq \frac{2F_s l}{SL}$ to open a gap.

The torque at the hinge T_x^* can be expressed as the following governing equation [1,2], where one end is constrained and one end is under torsion.

$$\frac{\mathrm{E}}{1-\mu^2} I_w \theta''' - GJ_k \theta'' = 0 \tag{4}$$

where E is the elasticity modulus of the material, θ is the torsion angle, μ is the Poisson ratio, I_w $(=\frac{l_h^3 h^3}{144})$ and J_k $(=\frac{l_h h^3}{3})$ are the inertia moment and torsional rigidity of the cross section.

The geometry of the lever and the hinge is given in Fig. S2. By solving this equation, the torque at the hinge is found to be

$$T_x^* = \frac{K^3 \bar{E} I_w \arctan\left(\frac{t}{l}\right)}{w^3\left[K - Tanh[K]\right]} \tag{5}$$

where $K^2 = \frac{(1-\mu)144}{6}(\frac{w}{h})^2$ and $\bar{E} = \frac{E}{1-\mu^2}$.

From the above theoretical analysis, the expression of the critical pressure P_c can be

obtained, which determines the open and close state of the FAST-POCT platform. The analysis shows that the parameters L and t/l can prominently affect the critical pressure and can be used to tune its value.

Figure S1

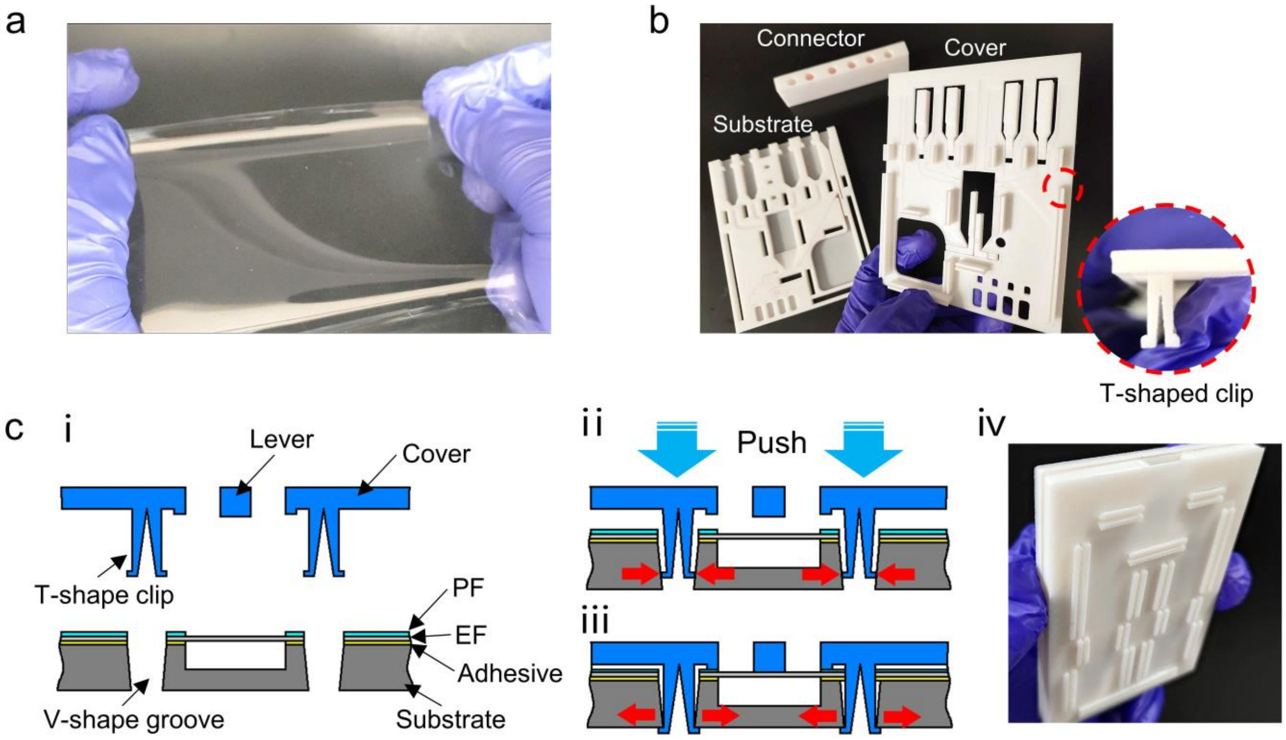

Figure S1. FAST-POCT platform components and illustration for assembly. a, Picture of the PDMS film. **b,** Picture of 3D printed substrate, cover and connector. The inset shows the details of the T-shape clip. **c,** Cross-sectional diagram for assembly, (i) T-shape clip on the cover, V-shape groove structure on the substrate, PF for plastic film, EF for elastic film. (ii) The T-shape clip has a clearance between two legs. When the clip is pushed into the groove, the two legs can slightly be bent and (iii) recover to its original state as it comes through the groove and fixes the cover and the substrate. (iv) Pictures of the back view of the device, which shows the assembled clips and grooves.

Figure S2

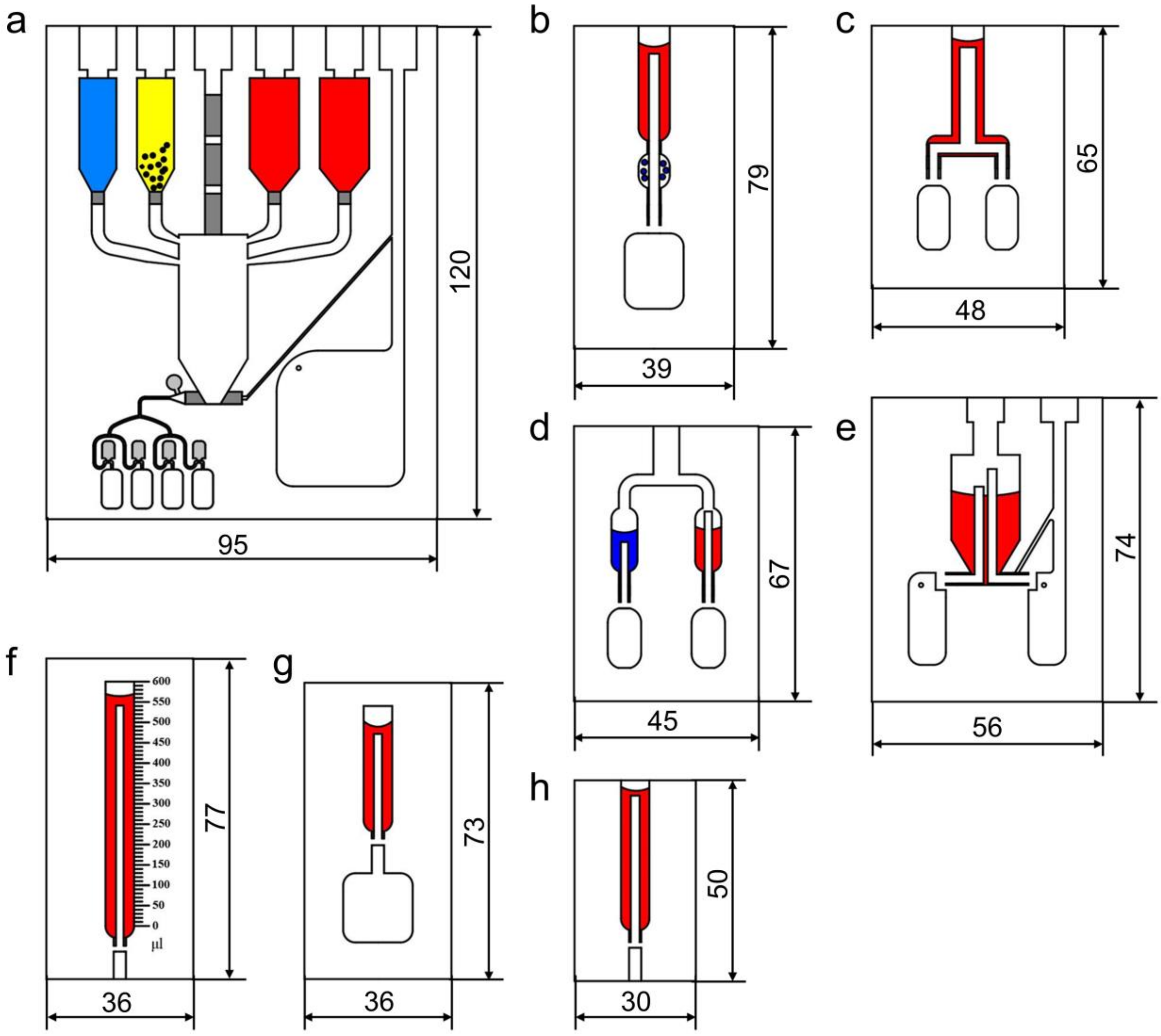

Figure S2. Schematic diagram for the dimensions of FAST-POCT. a, FAST-POCT PCR device. **b,** Cascaded device. **c,** Simultaneous device. **d,** Sequential device. **e,** Selective device. **f,** On-demand testing device. **g,** Robustness testing device. **h,** Long-term storage testing device. The thickness of all the devices is 7 mm.

Figure S3

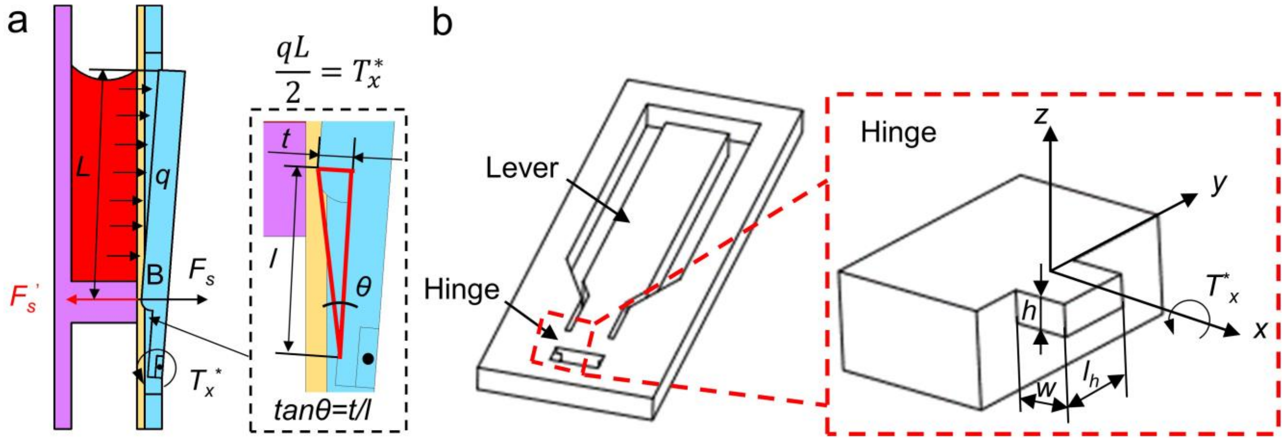

Figure S3. Schematic diagram for theoretical analysis of FAST. a, Cross-sectional diagram and force analysis of FAST. **b**, Schematic diagram of the theoretical analysis for the hinge structure, which can provide the sealed force and fast recover performance for the FAST-POCT platform.

Figure S4

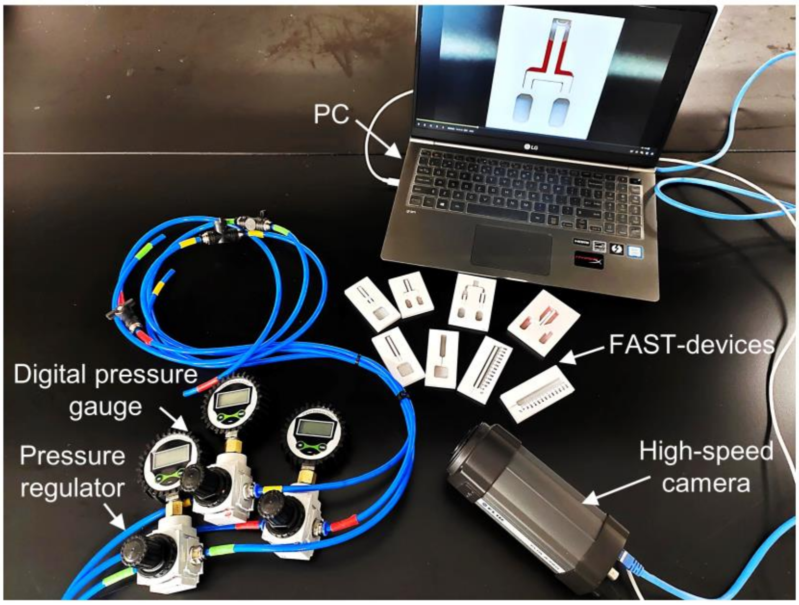

Figure S4. Picture of the testing system for the fluid flowing behavior. The system mainly comprises of digital pressure gauge, pressure regulators, an air compressor (not shown in this picture), a high-speed camera, the FAST-devices to be tested, and a PC. The digital pressure can provide an accurate pressure readout and the pressure can be tuned by the pressure regulator.

Figure S5

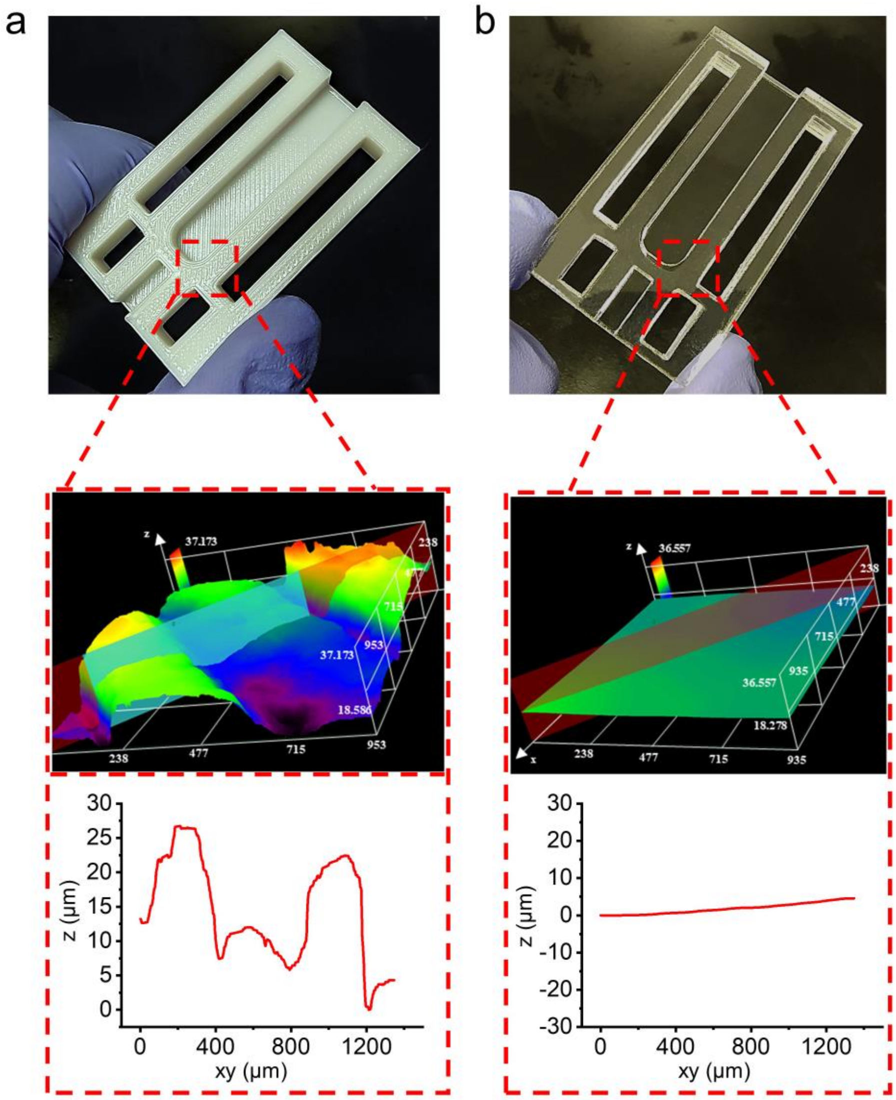

Figure S5. Images of the surface roughness comparison of a, 3D printing material and b, PMMA material.

Figure S6

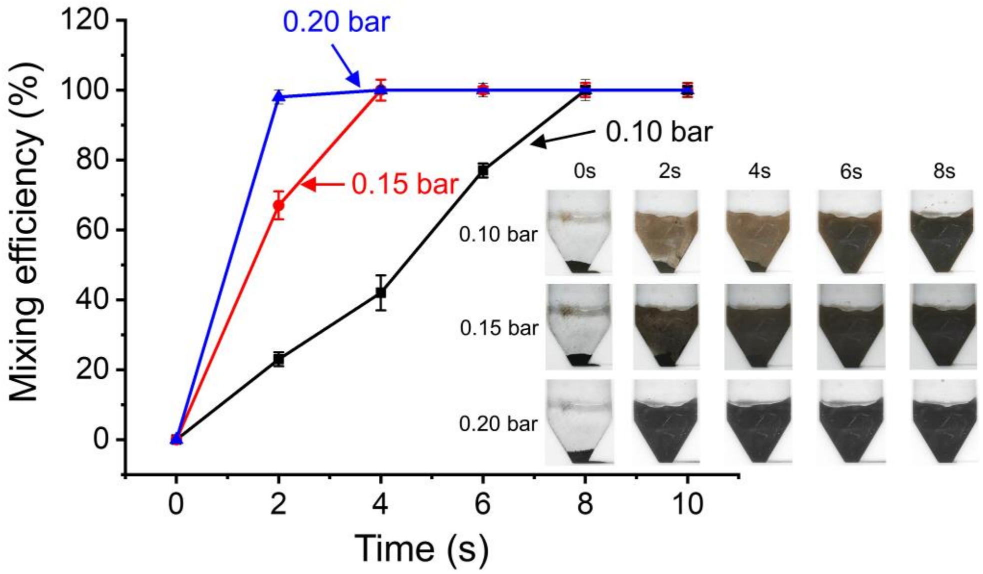

Figure S6. Bubble mixing performance. When 0.10 bar pressure was applied, it took 8 s to complete a full mixing. As a comparison, it took 2 s and 4 s for 0.20 bar and 0.15 bar pressure to obtain a full mixing, respectively. *n* = 6 independent experiments were conducted with the data shown as ± s.d. Source data are provided as a Source Data file.

Figure S7

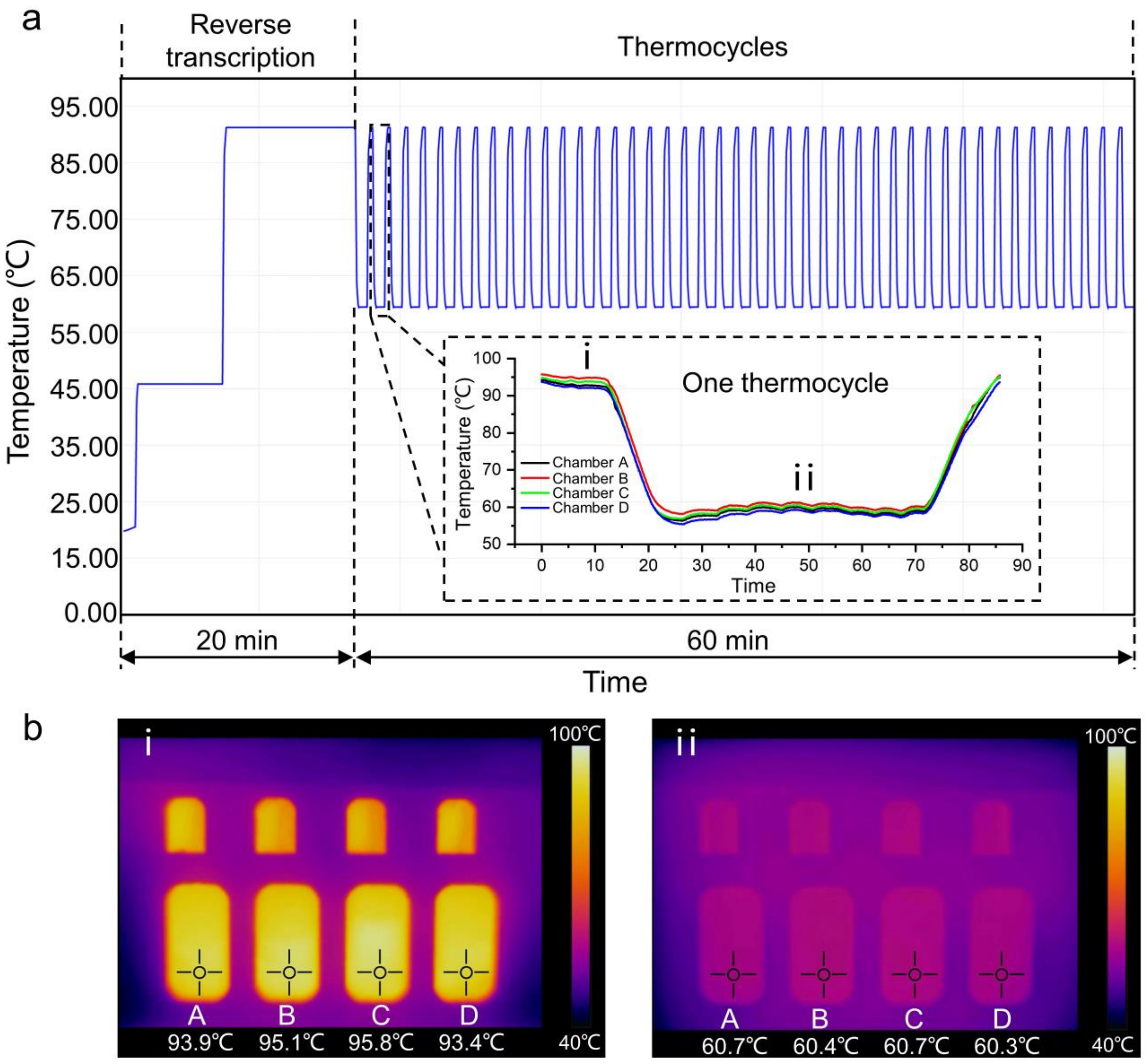

Figure S7. Temperature profile for the testing process. a, Reverse transcription time is 20 min. Thermocycling time is 60 min. One thermocycling time is 90 s. b, Thermal images during PCR testing for one thermocycle.

Figure S8

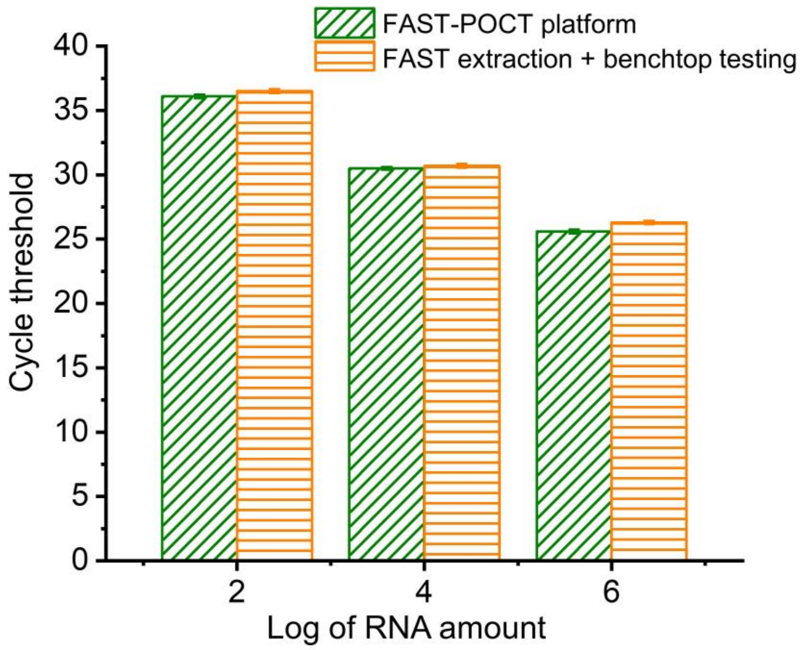

Figure S8. The results for IBV PCR testing using FAST-POCT platform and benchtop platform with FAST extracted RNA samples. *n* = 6 biologically independent experiments were conducted with the data shown as ± s.d. Source data are provided as a Source Data file.

Figure S9

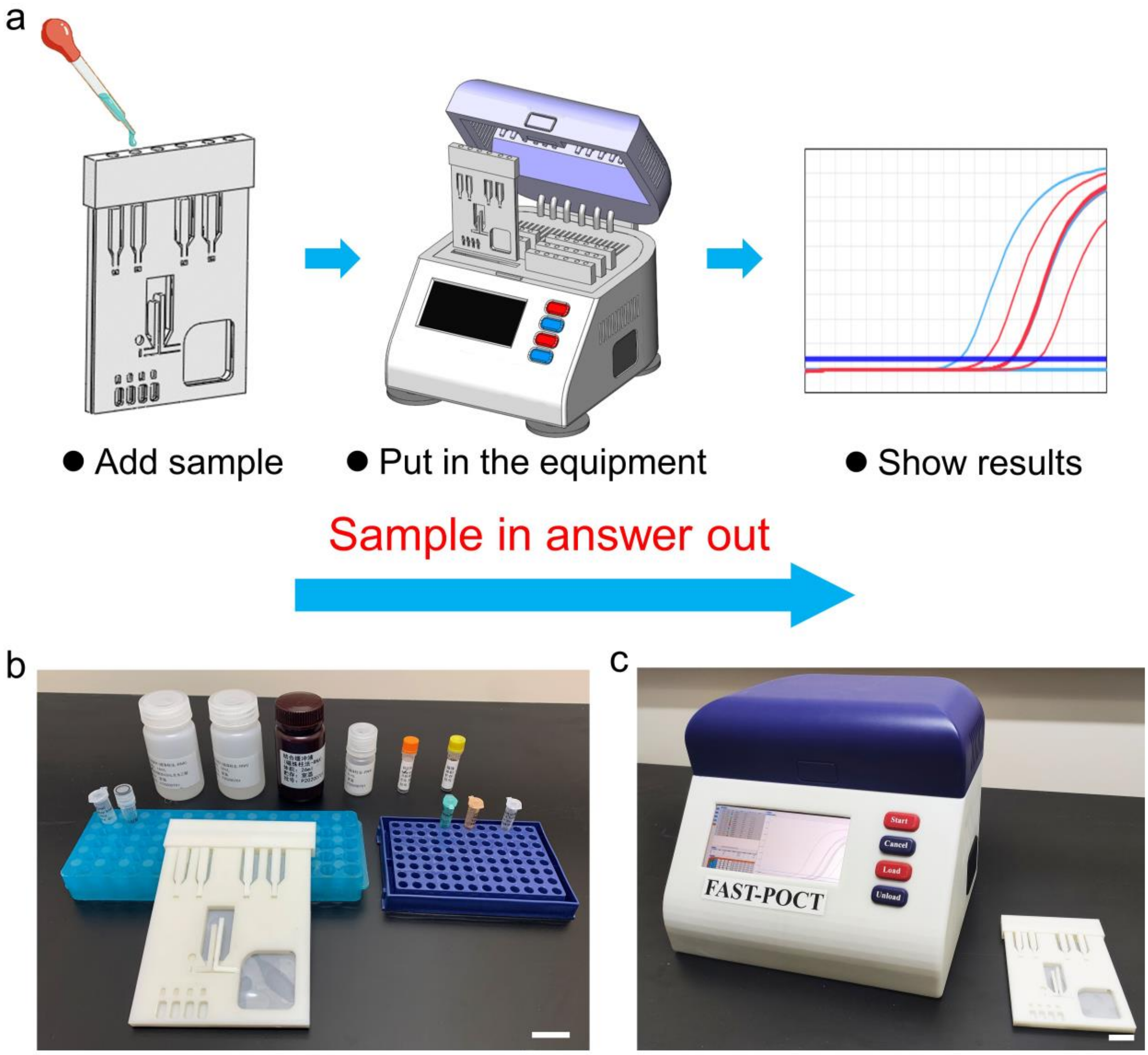

Figure S9. FAST-POCT instrument. a, Schematic diagram for "sample in answer out" performance of the FAST-POCT platform. The operator only needs to pipette the sample into the platform, insert the platform to the instrument and read the testing result after 82 min. The instrument was designed to include 6 separate testing modules and can handle up to 6 samples at once, possessing large-scale POCT potentials. **b,** Picture of the FAST-POCT platform and the reagents. **c,** Picture of the FAST-POCT instrument. All the scale bars, 1cm.

Figure S10

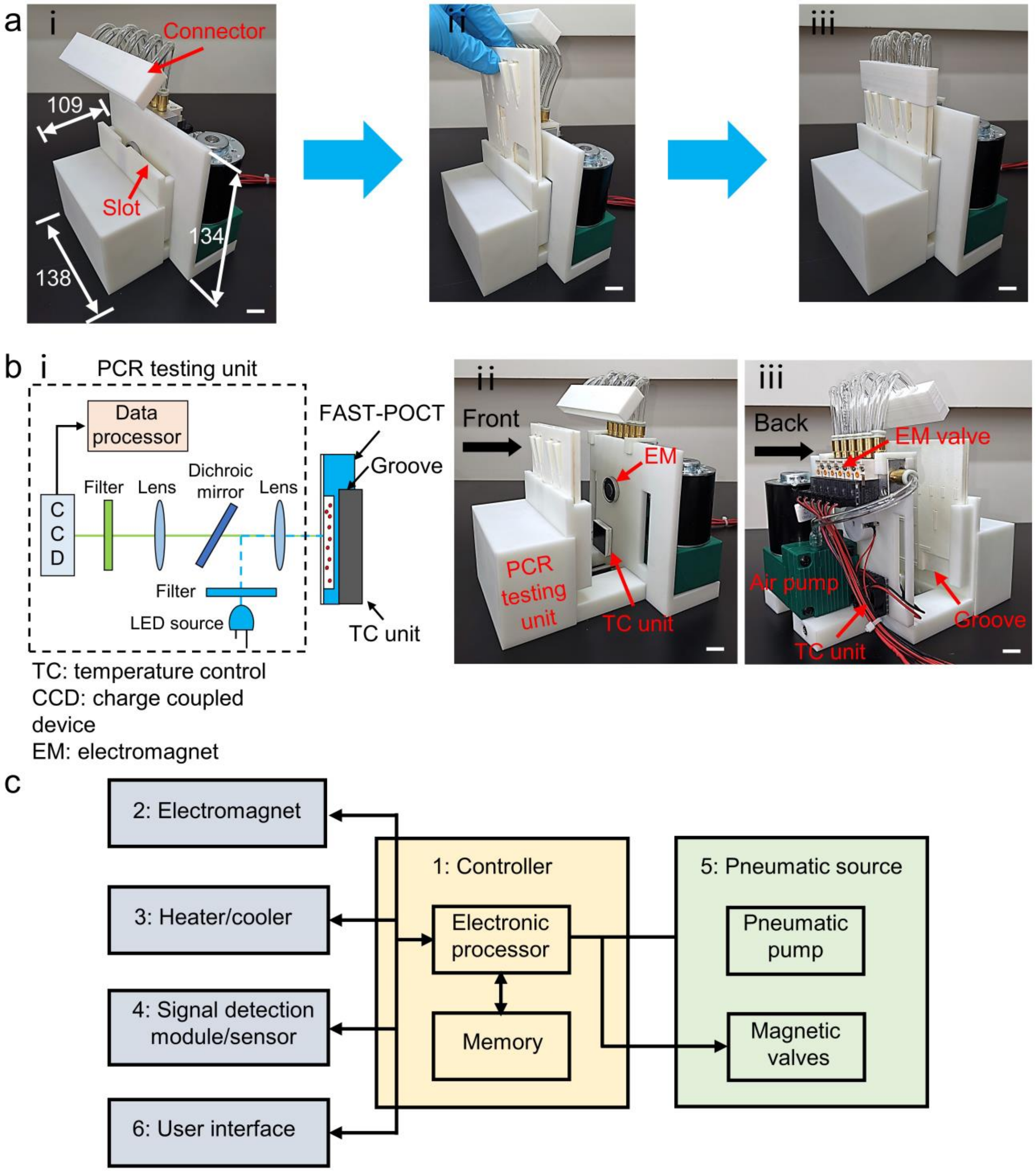

Figure S10. Illustration of the FAST-POCT testing module. a, The demonstration of the way to place the FAST-POCT device into a testing module prototype. **b,** i) The schematic diagram of the PCR testing unit. ii) Picture of the front view of the testing module prototype. iii) Picture of the back view of the testing module prototype. **c,** Flow chart of the relationship of different components. The scale bars are 10 mm.

Figure S11

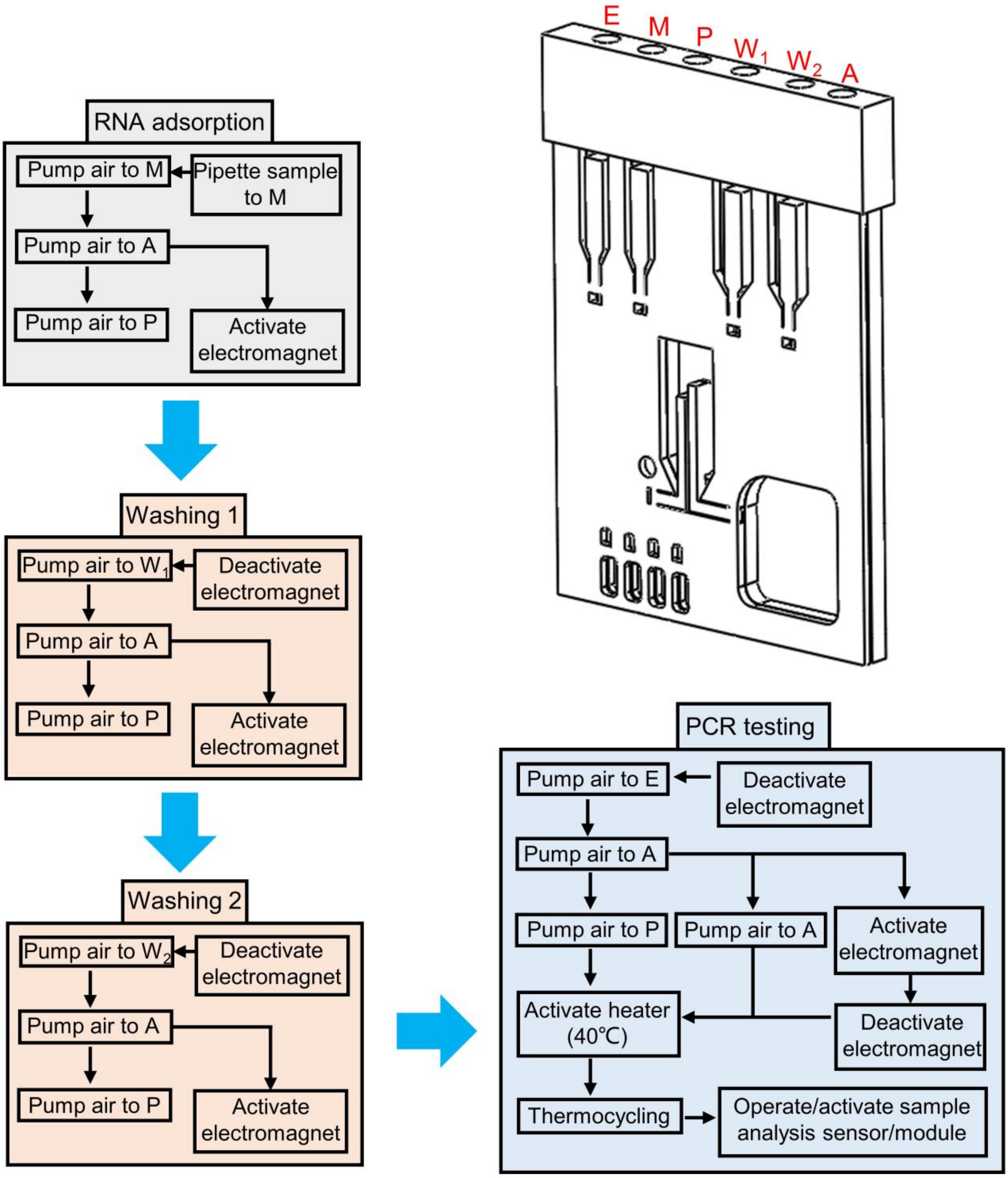

Figure S11. Flow chart of the working principles of the FAST-POCT instrument.

Figure S12

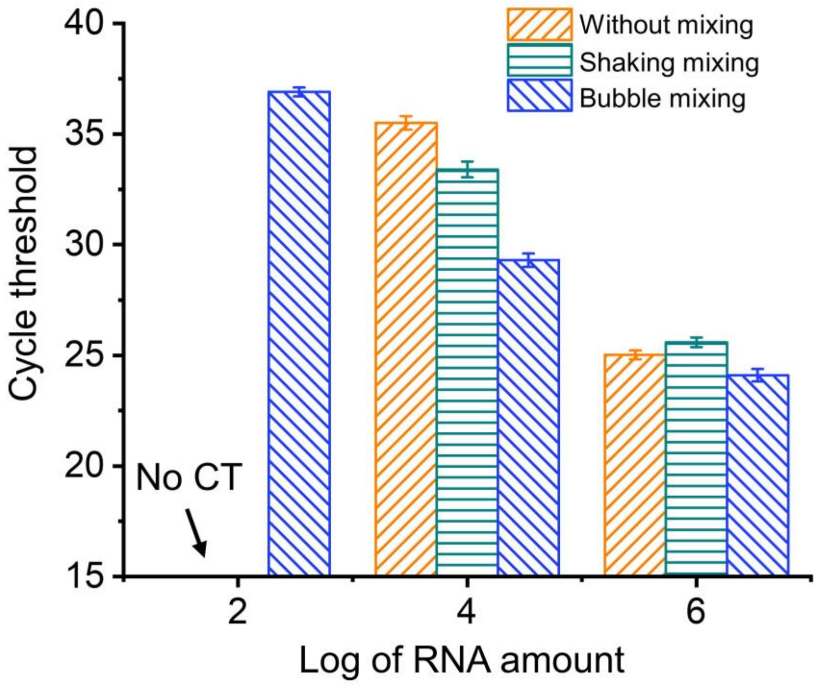

Figure S12. Comparison of PCR performance for shaking mixing, bubble mixing and without mixing as a control group. *n* = 6 biologically independent experiments were conducted with the data shown as ± s.d. Source data are provided as a Source Data file.

Figure S13

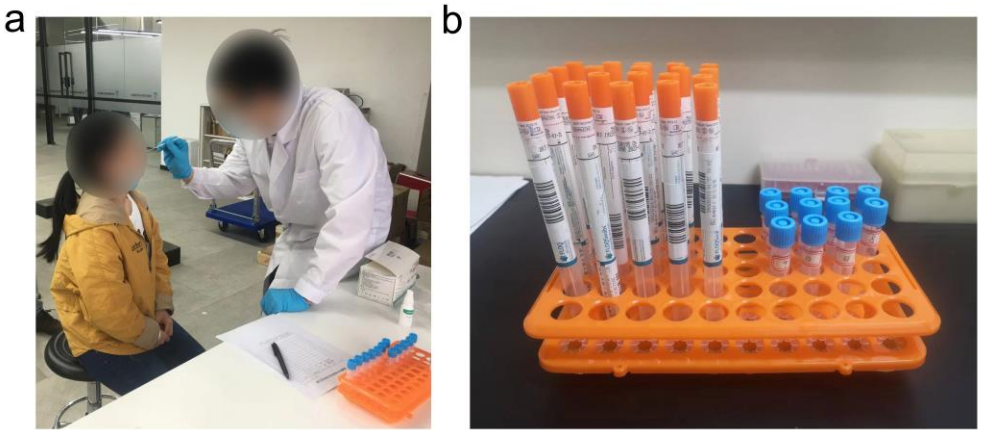

Figure S13. Pictures of a, nasal swab collection site and b, the collected nasal swab.

Supplementary Table 1. Material cost estimate of the FAST-POCT system.

Instrument (main components)

Description	Supplier	Size	Cost
Air pump	Zhirong, China<br>Air flow: 10L/min<br>Positive pressure: ＞1.5bar	L×W×H:<br>106×51×84 mm	$13.12
Electromagnetic valve	Highend, China<br>Working pressure: 0~7bar<br>Response time: 10ms	L×W×H:<br>82.5×39×25 mm	$33.41
Electromagnet	Elecall, China<br>Maximum force: 15kg	Φ30×22 mm	$3.13
Temperature control unit	Yexian,China<br>Accuracy: ±0.1℃	L×W×H:<br>40×40×25 mm	$123.25
PCR testing unit	Customized<br>Excitation: 470nm<br>Emission: 510nm	L×W×H:<br>50×37×32 mm	$75.47

Fast-POCT device (for one person)

Description	Supplier	Size	Cost
Substrate[1]	Ender, China	L×W×H:<br>120×95×5 mm	$0.28
Cover[1]	Ender, China	L×W×H:<br>120×95×2 mm	$0.19
Elastic film[2]	Dow Corning, Slygard 184, USA	L×W×H:<br>120×95×0.35 mm	$0.38
Plastic film	Saiweige, China	L×W×H:<br>120×95×0.1 mm	$0.01
Adhesive film	Adhesive research, 90880, USA	L×W×H:<br>120×95×0.14 mm	$0.47
Purification kit[3]	Liferiver, Z-ME-0010, China	N/A	$1.25
Testing kit[3]	Liferiver, RR-0051-02/RR-0052-02, China	N/A	$2.62
Estimated total material cost of a FAST-POCT kit for multiplexed influenza tests			$5.20

[1] The substrate and cover are fabricated using 3D printing techniques in the PCR testing experiments and the cost can be reduced in the future when the mass-production techniques are applied like mold-injection.

[2] The elastic film used in the experiments are made from Slygard 184 using mold-casting techniques. Further reduction of the cost should be possible in the future if commercially available elastic films are applied.

[3] The purification and testing kits are purchased from supplier for experimental purposes. When the FAST-POCT device is in the market and the kit can be purchased in a large scale, the kit cost can be further reduced.

Supplementary Table 2. Comparisons among some POCT PCR studies and technologies.

Detection performance						
Reference	FAST-POCT (this work)	Gzilwik, et al, Lab Chip, 15, 3749-3759 (2015)	Clime, et al, Lab Chip, 15, 2400-2411 (2015)	Wang, et al, Biosens. Bioelectron, 41, 484-491 (2013)	Rombach, et al, Analyst, 145, 7040-7047 (2020)	Jung, et al, Biosens. Bioelectron, 68, 218-224 (2015)
Target	Influenza RNA	Bacterial pathogens DNA	DNA	HIV DNA	Respiratory tract infection pathogens	Influenza A RNA
Method	RT-PCR	RT-PCR	Only DNA extraction	RT-PCR	RT-PCR	Isothermal PCR
Detection limit	15 copies	200 cfu of S. agalactiae	—[1]	62 copies	—	10 copies
Testing time	82 min	225 min	—	95 min	200 min	45 min
**Fluid handling capability**						
Multifunctional dispensing	Cascaded, simultaneous, sequential and selective dispensing	Inward dispensing	Inward dispensing	—	Inward dispensing	Two-way dispensing
On-demand releasing	Rapid and proportional to the applied pressure	—	Yes	Yes	—	—
Robust operation	No leaking under the vibration of 150 rad/min	—	—	—	—	—
Long-term storage	Accelerated life tests of DI water with weight loss less than 0.3% for two years	—	—	—	Yes	—
Liquid properties	Manipulating the liquid with viscosity as high as 5,500 cP	Viscosity of 16 cP with efficiency of 75%	—	—	—	—
Driving mechanism	Positive pressure	Centrifugal force	Centrifugal force and positive pressure	Negative pressure	Centrifugal force	Centrifugal force

[1] The dash "-" means that the related results or features were not demonstrated in the paper.

Supplementary Table 3. Clinical sample information.

Patients			Control individuals		
No.	Age	Sex	No.	Age	Sex
1	36	M	19	26	M
2	34	F	20	33	F
3	22	F	21	38	F
4	27	F	22	40	M
5	41	M	23	33	F
6	45	M	24	26	F
7	27	F	25	35	M
8	25	F	26	27	F
9	54	F	27	25	M
10	38	F	28	22	M
11	27	M	29	27	F
12	37	F	30	45	M
13	52	M	31	36	M
14	47	M	32	33	M
15	29	M	33	28	F
16	30	F	34	27	F
17	31	M	35	29	M
18	33	M	36	32	M

Supplementary References

1. S. P. Timoshenko and J. N. Goodier. Theory of elasticity[M]. MCGRAQ-HILL, 1970.
2. V. Z. Vlasov. Thin-walled elastic beams, second edition[M]. Jerusalem: Israel program for scientific transactions, 1961.
