# Supplementary material for: A film-lever actuated switch technology for multifunctional, on-demand, and robust manipulation of liquids
Source: Nat Commun. 2022 Aug 20;13:4902. doi: 10.1038/s41467-022-32676-4 (PMC9391643; doi:10.1038/s41467-022-32676-4)
Supplement: Supplementary file 3 — Description of Additional Supplementary Files [file 41467_2022_32676_MOESM3_ESM.pdf]

Description of Additional Supplementary Files

File Name: Supplementary Movie 1
Description: Demonstration of the lever movement of the FAST-POCT platform.

File Name: Supplementary Movie 2
Description: Multifunctional dispensing demonstration, including cascaded, simultaneous, sequential and selective dispensing modes.

File Name: Supplementary Movie 3
Description: Robustness test video using a capillary-based device as a comparison.

File Name: Supplementary Movie 4
Description: On-demand releasing test video using a capillary-based device as a comparison.

File Name: Supplementary Movie 5
Description: Flow behavior testing video with the liquids of different properties.

File Name: Supplementary Movie 6
Description: Working procedure video of the FAST-POCT platform.

File Name: Supplementary Movie 7
Description: Thermal imaging video for one thermocycle.

File Name: Supplementary Movie 8
Description: Wax sealing process of the FAST-POCT platform using a non-wax device as a comparison.
