# Supplementary material for: A film-lever actuated switch technology for multifunctional, on-demand, and robust manipulation of liquids
Source: Nat Commun. 2022 Aug 20;13:4902. doi: 10.1038/s41467-022-32676-4 (PMC9391643; doi:10.1038/s41467-022-32676-4)
Supplement: Supplementary file 12 — Reporting Summary [file 41467_2022_32676_MOESM12_ESM.pdf]

nature portfolio

Corresponding author(s): Hanqing Jiang

Last updated by author(s): Aug 8, 2022

# Reporting Summary

Nature Portfolio wishes to improve the reproducibility of the work that we publish. This form provides structure for consistency and transparency in reporting. For further information on Nature Portfolio policies, see our Editorial Policies and the Editorial Policy Checklist.

## Statistics

For all statistical analyses, confirm that the following items are present in the figure legend, table legend, main text, or Methods section.

| n/a | Confirmed | |
|---|---|---|
| ☐ | ☒ | The exact sample size (*n*) for each experimental group/condition, given as a discrete number and unit of measurement |
| ☐ | ☒ | A statement on whether measurements were taken from distinct samples or whether the same sample was measured repeatedly |
| ☐ | ☒ | The statistical test(s) used AND whether they are one- or two-sided<br>*Only common tests should be described solely by name; describe more complex techniques in the Methods section.* |
| ☒ | ☐ | A description of all covariates tested |
| ☒ | ☐ | A description of any assumptions or corrections, such as tests of normality and adjustment for multiple comparisons |
| ☐ | ☒ | A full description of the statistical parameters including central tendency (e.g. means) or other basic estimates (e.g. regression coefficient) AND variation (e.g. standard deviation) or associated estimates of uncertainty (e.g. confidence intervals) |
| ☒ | ☐ | For null hypothesis testing, the test statistic (e.g. *F*, *t*, *r*) with confidence intervals, effect sizes, degrees of freedom and *P* value noted<br>*Give P values as exact values whenever suitable.* |
| ☒ | ☐ | For Bayesian analysis, information on the choice of priors and Markov chain Monte Carlo settings |
| ☒ | ☐ | For hierarchical and complex designs, identification of the appropriate level for tests and full reporting of outcomes |
| ☐ | ☒ | Estimates of effect sizes (e.g. Cohen's *d*, Pearson's *r*), indicating how they were calculated |

*Our web collection on statistics for biologists contains articles on many of the points above.*

## Software and code

Policy information about availability of computer code

| | |
|---|---|
| Data collection | The data were collected via commercial or open-source software (SLAN-96P, FLIR ETS320, Olympus Fluorescence IX83). |
| Data analysis | The data were analyzed via commercial or open-source software (Image-Pro Plus, OriginPro 2021, Matlab R2010b, SLAN-96P, FLIR ETS320, Olympus Fluorescence IX83). |

For manuscripts utilizing custom algorithms or software that are central to the research but not yet described in published literature, software must be made available to editors and reviewers. We strongly encourage code deposition in a community repository (e.g. GitHub). See the Nature Portfolio guidelines for submitting code & software for further information.

## Data

Policy information about availability of data

All manuscripts must include a data availability statement. This statement should provide the following information, where applicable:

- Accession codes, unique identifiers, or web links for publicly available datasets
- A description of any restrictions on data availability
- For clinical datasets or third party data, please ensure that the statement adheres to our policy

The data supporting the results in this study are available in the Supplementary Information and the data used to make the figures can be found in "Source Data" excel file.

## Human research participants

Policy information about studies involving human research participants and Sex and Gender in Research.

| | |
|---|---|
| Reporting on sex and gender | All the findings are not applied to only one sex or gender. Clinical samples including 19 males and 17 females were collected and consent has been obtained for sharing of individual-level data. The detailed information about the sex or gender can be found in Table 3 in the Supplementary Information.  The sex or gender was not relevant to this study because no evidence indicates the infection of influenza has a relationship with the sex or gender. |
| Population characteristics | The clinical samples were from the First Affiliated Hospital of Zhejiang University (Hangzhou, Zhejiang). Detailed information about the samples is provided in Table 3 in the Supplementary Information. |
| Recruitment | Clinicians recruited subjects who presented with IBV symptoms or who were suspected of IBV infection. |
| Ethics oversight | The study was approved by the ethics committee of the First Affiliated Hospital of Zhejiang University (IIT20220330B). |

Note that full information on the approval of the study protocol must also be provided in the manuscript.

# Field-specific reporting

Please select the one below that is the best fit for your research. If you are not sure, read the appropriate sections before making your selection.

☒ Life sciences ☐ Behavioural & social sciences ☐ Ecological, evolutionary & environmental sciences

For a reference copy of the document with all sections, see nature.com/documents/nr-reporting-summary-flat.pdf

# Life sciences study design

All studies must disclose on these points even when the disclosure is negative.

| | |
|---|---|
| Sample size | 36 clinical samples (18 IBV positive and 18 IBV negative) were used in our study to validate FAST-POCT's clinical applicability. |
| Data exclusions | No data were excluded. |
| Replication | Conventional RT-PCR and FAST-POCT RT-PCR were validated by  six repeats per experiment. |
| Randomization | Randomization was not relevant to the study, because the main purpose of this study is to demonstrate a microfluidic manipulation method and to test the performance of the FAST-POCT RT-PCR against conventional RT-PCR. |
| Blinding | The investigator was not blinded to group allocation during the experiment. Blinding was not relevant because the main purpose of the study was to validate the performance of the FAST-POCT platform and all the results were confirmed and compared with the the gold standard conventional RT-PCR method. |

# Reporting for specific materials, systems and methods

We require information from authors about some types of materials, experimental systems and methods used in many studies. Here, indicate whether each material, system or method listed is relevant to your study. If you are not sure if a list item applies to your research, read the appropriate section before selecting a response.

## Materials & experimental systems

| n/a | Involved in the study | |
|---|---|---|
| ☒ | ☐ | Antibodies |
| ☒ | ☐ | Eukaryotic cell lines |
| ☒ | ☐ | Palaeontology and archaeology |
| ☒ | ☐ | Animals and other organisms |
| ☒ | ☐ | Clinical data |
| ☒ | ☐ | Dual use research of concern |

## Methods

| n/a | Involved in the study | |
|---|---|---|
| ☒ | ☐ | ChIP-seq |
| ☒ | ☐ | Flow cytometry |
| ☒ | ☐ | MRI-based neuroimaging |
